# Age-Related Effects of Stimulus Type and Congruency on Inattentional Blindness

**DOI:** 10.3389/fpsyg.2018.00794

**Published:** 2018-05-23

**Authors:** Han-Hui Liu

**Affiliations:** Department of Youth Work, China Youth University of Political Studies, Beijing, China

**Keywords:** attention, inattentional blindness, adolescents, young adults, middle-aged adults

## Abstract

**Background:** Most of the previous inattentional blindness (IB) studies focused on the factors that contributed to the detection of unattended stimuli. The age-related changes on IB have rarely been investigated across all age groups. In the current study, by using the dual-task IB paradigm, we aimed to explore the age-related effects of attended stimuli type and congruency between attended and unattended stimuli on IB.

**Methods:** The current study recruited 111 participants (30 adolescents, 48 young adults, and 33 middle-aged adults) in the baseline recognition experiments and 341 participants (135 adolescents, 135 young adults, and 71 middle-aged adults) in the IB experiment. We applied the superimposed picture and word streams experimental paradigm to explore the age-related effects of attended stimuli type and congruency between attended and unattended stimuli on IB. An ANOVA was performed to analyze the results.

**Results:** Participants across all age groups presented significantly lower recognition scores for both pictures and words in comparison with baseline recognition. Participants presented decreased recognition for unattended pictures or words from adolescents to young adults and middle-aged adults. When the pictures and words are congruent, all the participants showed significantly higher recognition scores for unattended stimuli in comparison with incongruent condition. Adolescents and young adults did not show recognition differences when primary tasks were attending pictures or words.

**Conclusion:** The current findings showed that all participants presented better recognition scores for attended stimuli in comparison with unattended stimuli, and the recognition scores decreased from the adolescents to young and middle-aged adults. The findings partly supported the attention capacity models of IB.

## Introduction

Inattentional blindness (IB) is the failure to consciously detect unattended, irrelevant events when observers are engaged in an attention-demanding task (Mack and Rock, 1998). This phenomenon is common in daily life, for example, when you have a conversation with your friends, you may neglect the waving hand of another friend, although he is indeed in your visual field. Sometimes the oversights may lead to traffic accidents or other serious consequences. In a simulated real-world assault, researchers found many participants did not notice the fight at night or in the day (Chabris et al., 2011).

Many studies investigated the influencing factors and mainly focused on two types of factors and their interactions (Gu et al., 2005; Cartwright-Finch and Lavie, 2007). The first kind of studies mainly concerned the external unattended stimuli attributes, including the size, color, location, speed, spatial distance, or similarity to the attended stimuli (Mack and Rock, 1998; Most et al., 2001, 2005; Koivisto et al., 2004; Karns and Rivardo, 2010; Graham and Burke, 2011). The second kind of studies focused on the internal factors such as domain-related skills of the participants (Memmert, 2006; Drew et al., 2013), attentional set of the observers to the primary task (Horwood and Beanland, 2016), and the perceptual load of the main task (Cartwright-Finch and Lavie, 2007; Simons and Jensen, 2009). Previous studies suggested that both the external factors and internal factors could contribute to the IB (Simons and Chabris, 1999), however, few age-related studies simultaneously investigated the external and internal factors.

Previous studies showed that similarity between attended and unattended stimuli would affect the detection rate. Participants could capture the unattended stimuli more easily when they were similar to the attended stimuli (Simons and Chabris, 1999; Most et al., 2001, 2005; Karns and Rivardo, 2010; Horwood and Beanland, 2016). However, the results were inconsistent. Neisser (1979) found the similarity would not influence the detection of unattended stimuli. These inconsistent results may attribute to different experimental paradigms. Neisser (1979) used transparent experimental video stimuli that different from later studies. More evidences were needed to clarify the effects of the similarity with differential experimental paradigms. Moreover, the age-related effects of similarity are also inconsistent. Based on the dynamic experimental paradigm used by Simons and Chabris (1999) and Graham and Burke (2011) found similarity did not result in detection differences between younger (17–22 years) and older adults (61–81 years). In their study, a black gorilla appeared unexpectedly during a passing basketball game. Observers were divided into two groups randomly, one group needed to count the number of passes made by the white team (low similarity condition), while the other were required to count the number of passes made by the black team (high similarity condition). The results showed both young and older adults were more likely to detect unattended stimulus under high similarity condition. In addition, Horwood and Beanland (2016) had also explored the effect of age and color similarity on attention capture and did not find the interactions between them. However, other researchers revealed that the similarity to the attended stimuli was more helpful in allowing older adults to detect the unattended stimuli (Carlson et al., 1995). In that study, distracting materials was randomly interspersed amidst the target text, as the distractors (words or short terms) becoming more similar to the target text, it could capture more attention. Compared with young adults, older adults were distracted more by these unattended stimuli. These findings suggested that age might influence the detection of unattended stimuli, and more studies were warranted to further clarify the age-related effects of the similarity on IB.

Although many studies investigated the influencing factors of IB and tried to reveal its underlying mechanisms, few studies studied the age-related changes on IB. Graham and Burke (2011) recruited 31 young adults and 26 older adults and tried to find the age-related IB rate with a dynamic IB paradigm developed by Simons and Chabris (1999), and they found that older adults showed higher IB rate than young adults. In another research, Horwood and Beanland (2016) found an 89% IB rate in older adults and only 5% IB rate in young adults with static task. Similarly, in the dynamic task, they found a 38% rate of IB in older adults and an 8% rate of IB in young adults. A recent research across adult lifespan (18–75 years) used the online data collection approach to explore the age-related effects of IB within the 515 participants (Stothart et al., 2015), the logistic regression analyses showed that the probability of noticing the unattended object decreased with age, and the increase of 10 years of age was associated with 1.30-fold increase of IB. These results suggested that the IB rate increased with age from young to older adults. However, because of no previous studies simultaneously included the adolescents, therefore it was unknown whether these findings could extend to the adolescents. Moreover, contrary to the relatively consistent findings in adults, the age-related IB effects were inconsistent in the adolescents, Remington et al. (2014) found decreased IB rate with participants aged from 7 to 14 years old with the classical static IB paradigm. However, Zhang et al. (2017) investigated the IB rate in 7–14 years old participants with the dynamic IB paradigm, and they found no significant developmental difference in IB rate. The differential IB paradigms used in these two studies might contribute to the inconsistent results. These aforementioned findings implied that including adolescents and the adults simultaneously could better explore the age-related IB effects.

There were two types of cognitive aging models of attention to explain the age-related IB effects. According to attentional capacity model, attention was finite and the attentional capacity would decrease with increasing age, therefore less attentional capacity were left to process the unattended stimuli at conscious awareness level in older adults in comparison with young adults. This model predicted that older adults would show lower recognition score than young adults (Kahneman, 1973; Graham and Burke, 2011; Stothart et al., 2015). However, under inhibitory deficit model, older adults had deficits to prevent irrelevant stimuli from accessing conscious awareness, and could notice more of the unattended stimuli, thereby exhibiting higher recognition score than young adults (Connelly et al., 1991; Rowe et al., 2006; Kim et al., 2007). These two theories made opposite predictions for age differences in IB. Based on previous studies, Graham and Burke (2011) indicated that age-related deficits in inhibition of distraction did not occur at the level of explicit conscious attention, calling for revision of the emphasis on age differences in inhibitory control of access to consciousness.

Most of the previous IB studies used the static paradigm and dynamic paradigm to study IB, but we still needed more proofs of age-related IB effects based on other paradigm. Rees et al. (1999) developed a new IB paradigm to explore it. In their study, participants are told to observe the rapid stream of green letter strings (meaningful familiar words or meaningless strings of random consonants) superimposed on a rapid stream of red pictures. Participants needed to attend only the stream of green words or stream of superimposed red pictures and count the immediate repetition of the stimuli. The results showed that even when participants looked directly, no significant brain activation differences during the time course of foci of activity in the left frontal cortex and left temporal cortex were found between meaningful words and random letters. Therefore, researchers concluded that participants were blind to properties of the unattended words, which provided evidence that the paradigm was exactly an IB paradigm rather than an inattentional amnesia paradigm.

According to the aforementioned studies, most previous studies only focused on children, or investigated the IB rates between young and old adults. In the current study, we aimed to extend the age groups and included adolescents, young adults, and middle-aged adults to explore the age-related IB effects. There were no consistent dividing criterions for age stages. According to previous classification (Lin, 1995; Chan et al., 2014; Papalia and Feldman, 2015), adolescents were thought to begin at 10–13 years old and end at 18–20 years old, participants between 9 and 17 years were classified as adolescents in the current study (only one 9 years old child, the results were same when excluding this participant). Adulthood began from 18 to 20 years old, and ended at 60–64 years old, due to the large span of age range of adulthood (18–64 years old), and several lifespan studies found decreased cognitive performances from 18 to 20 years old (Salthouse, 2010; Hartshorne and Germine, 2015), in the current study, participants between 18 and 34 years older were divided as young adults, while participants between 35 and 64 years old were considered as middle-aged adults.

Moreover, because of the effects of similarity were inconsistent in previous studies, more evidences with differential experimental paradigm were warranted to further clarify the similarity on IB. Additionally, the pictures might be processed differently from the words (Walker et al., 2017). Therefore, in the current study, we aimed to explore whether the similarity and attended stimuli (words or pictures) would influence the recognition accuracy of unattended stimuli with the dual-task experimental paradigm. We hypothesized that the level of awareness for the irrelevant distracter would decreased with age, that is, more IB would be found in the middle-aged adults compared with the adolescents and young adults. Furthermore, we hypothesized that the congruency between unattended and attended stimuli would result in higher recognition scores in comparison with incongruency. Finally, we hypothesized that participants would show higher recognition scores when the unattended stimuli were figures.

## Materials and Methods

The study consisted of two parts: baseline recognition test and IB experiment. The former was used as the baseline recognition score to compare with the recognition scores during the IB experiments.

### Participants

The experiments included baseline recognition test and IB test. I chose different sets of participants in both IB and baseline experiment.

#### Baseline Recognition Test

One hundred and eleven participants participated in the baseline recognition test. Details of the participants’ information could be found in **Table 1**. Written informed consents were obtained from participants aged above 18 years old in accordance with the Declaration of Helsinki prior to the study; for participant under 18, both parents and children/adolescents signed informed consent forms. The study was approved by the local ethics committee of the department of Youth Work, China Youth University of political studies.

**Table 1:** Demographic information for participants in each group.

			Adolescents		Young adults		Middle-aged adults	
	Group		*M*	*SD*	*M*	*SD*	*M*	*SD*
N	Baseline recognition test		30		48		33	
	IB test	Repetitive pictures	72		74		34	
		Repetitive words	63		61		37	
Age (year)	Baseline recognition test		14.03	1.67	23.10	1.21	54.97	7.91
	IB test	Repetitive pictures	13.31	2.24	23.64	5.07	51.26	8.80
		Repetitive words	13.67	2.31	24.23	5.31	50.27	8.91
Female’ s ratio (%)	Baseline recognition test		53.3		70.8		66.7	
	IB test	Repetitive pictures	58.3		59.5		64.7	
		Repetitive words	66.7		65.6		48.6	

*N, sample number; IB, inattentional blindness.*

#### Inattentional Blindness Experiment

In total, 341 participants participated in the IB experiment; they were divided into two groups based on the main tasks. Participants with different age groups (adolescents, young adults, and middle-aged adults) were assigned into repetitive pictures group and repetitive words group. The age range of three age groups were same as the baseline recognition test. Other details can be found in **Table 1**.

The *t*-test revealed that no significant age differences were found between repetitive pictures and repetitive words group in adolescents (*t* = -0.92, *p* > 0.05, *d* = -0.16), young adults (*t* = -0.66, *p* > 0.05, *d* = -0.11), or middle-aged adults (*t* = 0.47, *p* > 0.05, *d* = 0.11). There were also no significant gender differences between repetitive pictures and repetitive words group in adolescents (χ^2^ = 0.99, *p* > 0.05, *d* = 0.17), young adults (χ^2^ = 0.53, *p* > 0.05, *d* = 0.13), or middle-aged adults (χ^2^ = 1.86, *p* > 0.05, *d* = 0.33).

### Materials

All the pictures in this study were selected from the standardized pictures set (Snodgrass and Vanderwart, 1980). The materials were divided into the baseline recognition test and the IB experiment.

The baseline recognition tests included the words and pictures recognition tests. During the words and pictures recognition test, participants were presented 32 words, while in the recognition test, we added 16 new words or pictures, and therefore, 48 words and pictures were presented in the recognition test. All the words were in green and Chinese characters that were presented in the center of the screen, the background was white, and the visual angle for words was 0.5°. All the pictures were printed in red and presented in the center of the screen, the background was white, the visual angle was 5°.

The IB experimental materials were words superimposed on the pictures. During the experiment, participants were required to only attend to the stream of words or only attend to the superimposed stream of pictures. The main task for the participants was to count how many repetitive words or pictures in the stream. To make sure of the level of difficulty for the main task, we set different angles for the repetitive pictures. If the first one was upright, then the second would be set 30° clockwise or counterclockwise. There were seven repetitive pictures or words.

All the procedures were presented with E-prime and were presented in the 17″ LCD, the resolution was 1024 × 768, and the refresh rate was 90 Hz.

### Experimental Design

The present study was a three-factor (attended stimuli type: attend words, attend pictures; congruency between attended and unattended stimuli: congruency and incongruency; age: adolescents, young adults, and middle-aged adults) mixed design.

**FIGURE 1:**
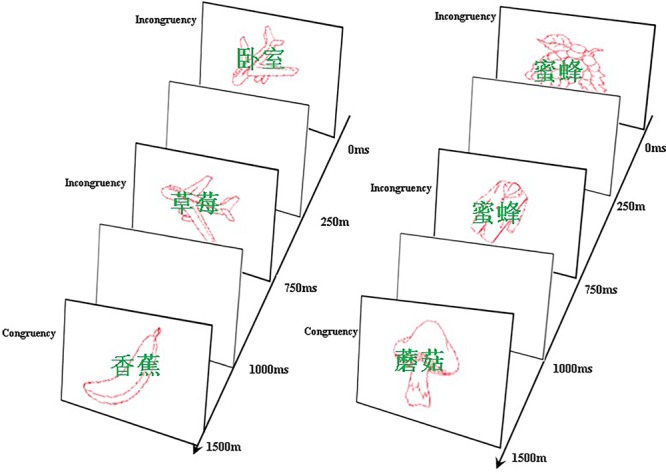
The left panel was schematic illustration of stimulus configuration in the repetitive pictures condition, attended pictures served as the target in the main task (e.g., airplane) while superimposed words were the unattended items. The right panel was schematic illustration of stimulus configuration in the repetitive words condition, attended words served as the target in the main task (e.g., 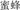) while superimposed pictures were the unattended items. 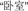 indicates bedroom, 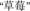 indicates strawberry, 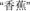 indicates banana, 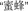 indicates bee, 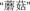 indicates mushroom.

The attended stimuli type and age were between-subject factors. In the IB experiment, the stimuli were words superimposed on pictures, participants across all age groups were divided into two groups randomly, half participants were required to only attend to the stream of words, their main task was to count the repetitive words during the stream of the stimuli, this group was named “repetitive words group.” The other half participants were required to only attend to the stream of pictures, the main task was to count the repetitive pictures during the stream of the stimuli, this group was called “repetitive pictures group.” For the “repetitive words group,” the words were the attended stimuli, and the pictures were the unattended stimuli. In the contrary, for the “repetitive pictures group,” the words were the attended stimuli, while the pictures were the unattended stimuli (**Figure 1**).

The congruency between an unattended stimuli and an attended stimuli was a within-subject factor. For half of the superimposed stimuli, the representation of words and pictures were congruent, while ur conditions for each participant: attended pictures, congruent; attended pictures, incongruent; attended words, congruent; attended words, the representation was incongruent.

### Procedures

#### Baseline Recognition Procedures

Take the word baseline recognition test, for example. Participants were required to remember the presented words. Each of the words presented for 250 ms in the center of the screen, then followed with 500 ms blank screen; in total, 32 words were presented. In the recognition phase, the 32 old words and 16 new words were presented, and participants were asked to judge whether they had seen the words or not. The picture baseline recognition test was the same as the words recognition test.

#### IB Procedure

The main task was to count the repetitive pictures or words during the stream of the stimuli. The seven repetitive pictures or words were presented one after another, and appeared every three or four streams. Each superimposed stimulus was presented for 250 ms, followed with 500 ms white background screen, and then the next stimulus. After the end of the presentation, participants were required to report how many repetitive pictures or words they attended, followed with the recognition test. If the participants’ main task was to count the repetitive words, then the recognition test assessed whether they had seen the pictures, and vice versa. The recognition test included 48 words/pictures, of which 32 were old, and 16 were new. For each participant, the recognition score in the recognition memory test for words or pictures was the index of the IB, higher score meant less IB; and it was calculated by hit rate minus false alert rate. For each group (attended pictures, congruent; attended pictures, incongruent; attended words, congruent; attended words, incongruent) in IB experiment, recognition accuracies also meant the recognition score and the index of IB.

## Results

### Baseline Recognition Test

**FIGURE 2:**
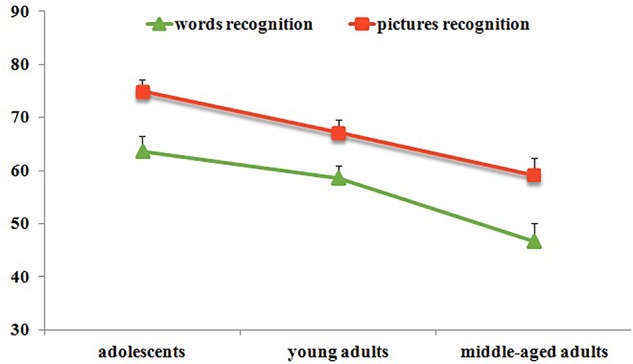
Recognition accuracy across all participants in baseline test.

The baseline recognition test results could be found in **Figure 2** and **Table 2**. The repeated measures ANOVA found that the age main effect was significant [*F*(2,108) = 11.84, *p* < 0.001, $\eta_{p}^{2}$ = 0.18]. Both adolescents and young adults performed better than middle-aged adults (*M*_D_ = 16.35, *p* < 0.001, *d* = 1.21; *M*_D_ = 9.90, *p* < 0.01, *d* = 0.71), while the adolescents performed significantly better than young adults (*M*_D_ = 6.45, *p* < 0.05, *d* = 0.50). The main effect of type of the recognition materials was significant [*F*(1,109) = 31.5, *p* < 0.001, $\eta_{p}^{2}$ = 0.23], the performance of the pictures recognition was higher than that of the words (*M*_D_ = 10.50). The interaction between materials type and age was not significant [*F*(2,108) = 0.41, *p* > 0.05, $\eta_{p}^{2}$ = 0.01].

**Table 2:** Recognition accuracy across all participants in baseline and IB experiment.

				Adolescents		Young adults		Middle-aged adults	
Test	Task	Group		*M*	*SD*	*M*	*SD*	*M*	*SD*
Baseline recognition test	Recognition for attended stimuli	Words recognition		63.65	15.69	58.53	17.02	46.69	19.42
		Pictures recognition		74.93	12.23	67.15	16.61	59.19	18.22
IB test	Recognition for	CY	Repetitive pictures	39.53	17.45	28.44	21.46	10.37	19.10
	unattended stimuli		Repetitive words	33.18	15.94	25.45	20.68	19.48	20.75
		ICY	Repetitive pictures	15.58	14.86	7.35	17.62	5.29	16.98
			Repetitive words	15.30	18.20	8.54	18.58	12.52	18.99

*CY, congruency group; ICY, incongruency group; IB, inattentional blindness.*

### IB Experiment Results

#### The Main Task Results in IB Experiment

**FIGURE 3:**
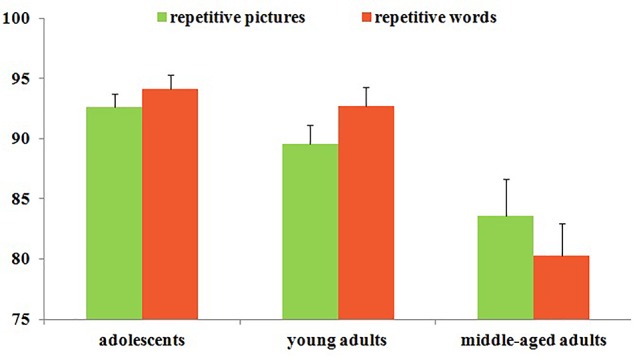
The main task accuracy across all participants.

The detection rates for repetitive pictures or words during the main task were presented in **Figure 3** and **Table 3**. The ANOVA showed that the age main effect was significant [*F*(2,338) = 19.56, *p* < 0.001, $\eta_{p}^{2}$ = 0.11]. The *post hoc* test revealed that both adolescents and young adults showed significantly higher detection rates than the middle-aged adults (*M*_D_= 11.44, *p* < 0.001, *d* = 0.91; *M*_D_= 9.11, *p* < 0.001, *d* = 0.63), and no significant differences were found between adolescents and young adults (*M*_D_= 2.33, *p* > 0.05, *d* = 0.20). The attended stimuli main effect was not significant [*F*(1,339) = 0.09, *p* > 0.05, $\eta_{p}^{2}$ = 0.00], no significant detection rates differences were found for attended words or pictures. Moreover, the interactions between age and attended stimuli were not significant [*F*(2, 338) = 1.52, *p* > 0.05, $\eta_{p}^{2}$ = 0.01].

**Table 3:** The main task accuracy across all participants.

	Adolescents		Young adults		Middle-aged adults	
	*M*	*SD*	*M*	*SD*	*M*	*SD*
Repetitive pictures	92.66	9.28	89.58	13.64	83.61	17.63
Repetitive words	94.10	9.82	92.74	11.84	80.31	16.27

#### The Recognition Results for Unattended Pictures and Words

The recognition accuracy performances were presented in **Table 2**. The repeated measures ANOVA revealed that the congruency main effect was significant [*F*(1,339) = 192.48, *p* < 0.001, $\eta_{p}^{2}$ = 0.37], suggesting that participants detected the unattended stimuli more when they had the same meaning with the attended stimuli. The main effect of age was significant [*F*(2,338) = 20.84, *p* < 0.001, $\eta_{p}^{2}$ = 0.11]; *post hoc* tests presented that adolescents showed higher recognition accuracy than young adults (*M*_D_= 8.52, *p* < 0.001, *d* = 0.55) and middle-aged adults (*M*_D_= 13.92, *p* < 0.001, *d* = 0.94), while the recognition accuracy in young adults was higher than middle-aged adults (*M*_D_= 5.40, *p* < 0.05, *d* = 0.32). The main effect of attended stimuli type was not significant [*F*(1,339) = 0.554, *p* > 0.05, $\eta_{p}^{2}$ = 0.002], and no significant recognition accuracy differences were found for attended words or pictures.

**FIGURE 4:**
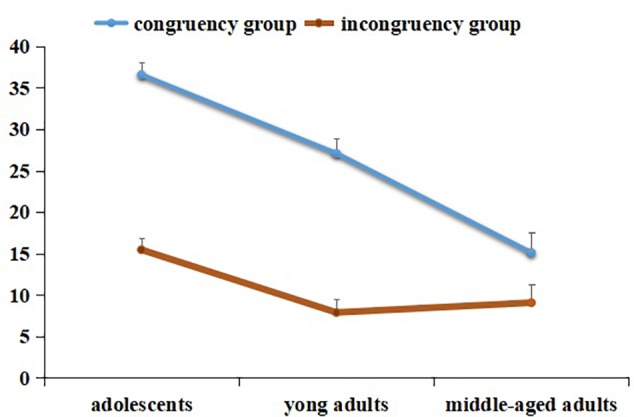
The interaction between age and congruency in IB experiment.

The interactions between congruency and age were significant [*F*(2,338) = 14.75, *p* < 0.001, *$\eta_{p}^{2}$*= 0.08]. The simple effect test showed that participants across all age groups presented higher recognition accuracy in congruency than representation incongruency [adolescents: *F*(1,133) = 158.52, *p* < 0.001, *d* = 1.17; young adults: *F*(1,133) = 130.98, *p* < 0.001, *d* = 1.09; middle-aged adults: *F*(1,69) = 6.86, *p* < 0.01, *d* = 0.33]. The simple effect test further showed that adolescents showed higher recognition accuracy than young adults (*M*_D_= 9.48, *p* < 0.001, *d* = 0.49) and middle-aged adults (*M*_D_= 21.45, *p* < 0.001, *d* = 1.18) under congruency, and the recognition accuracy in young adults was higher than middle-aged adults (*M*_D_= 11.97, *p* < 0.001, *d* = 0.57). Under incongruency, adolescents showed higher recognition accuracy than young adults (*M*_D_= 7.56, *p* < 0.001, *d* = 0.44) and middle-aged adults (*M*_D_= 6.39, *p* > 0.05, *d* = 0.37), and there were no significant differences between young adults and middle-aged adults (*M*_D_ = 1.17, *p* > 0.05, *d* = 0.06) (**Figure 4**).

**FIGURE 5:**
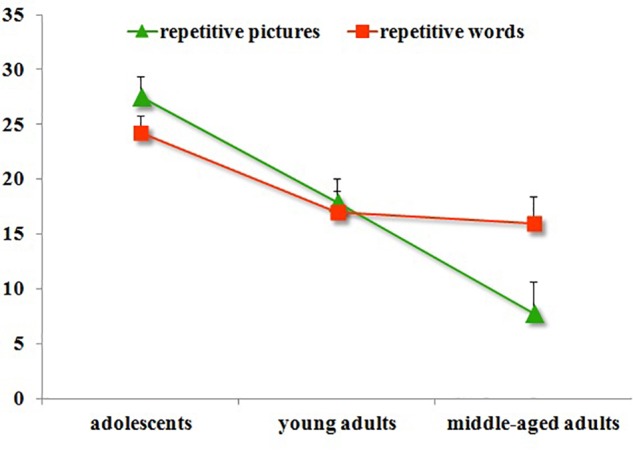
The interaction between age and stimuli type in IB experiment.

The interactions between age and attended stimuli type were significant [*F*(2,338) = 3.24, *p* < 0.05, $\eta_{p}^{2}$ = 0.02] (**Figure 5**). We therefore analyzed the recognition accuracy according to attended words and attended pictures. When participants attended pictures, analysis of the interactions with simple effects test showed that adolescents presented higher recognition accuracy than young adults (*M*_D_ = 9.66, *p* < 0.001, *d* = 0.63) and middle-aged adults (*M*_D_= 19.72, *p* < 0.001, *d* = 1.46), while young adults presented higher recognition accuracy than middle-aged adults (*M*_D_ = 10.07, *p* < 0.01, *d* = 0.63). When participants attended words, adolescents presented higher recognition accuracy than young adults (*M*_D_ = 7.25, *p* < 0.05, *d* = 0.46) and middle-aged adults (*M*_D_ = 8.24, *p* < 0.05, *d* = 0.52), while no significant differences were found between young adults and middle-aged adults (*M*_D_ = 0.99, *p* > 0.05, *d* = 0.06). The simple effect test further found that middle-aged adults showed higher recognition accuracy for the unattended pictures than the unattended words, and the differences approached significance [*F*(1,69) = 3.69, *p* = 0.056, *d* = 0.46]. No significant differences were found between unattended pictures or words in adolescents [*F*(1,133) = 1.61, *p* > 0.05, *d* = 0.22] or young adults [*F*(1,133) = 2.13, *p* > 0.05, *d* = 0.25]. The interactions between congruency and attended stimuli were not significant [*F*(1,339) = 1.61, *p* > 0.05, $\eta_{p}^{2}$ = 0.005]. The three-way repeated measures ANOVA revealed that the interactions among congruency, attended stimuli type, and age were insignificant [*F*(2,338) = 0.99, *p* > 0.05, $\eta_{p}^{2}$ = 0.006].

### The Comparisons of Recognition Accuracy Between Baseline Recognition Test and the IB Experiment

The *t*-test found that recognition scores in the baseline test were significantly higher than the IB experiments across adolescents (pictures recognition: *t* = 16.40, *p* < 0.001, *d* = 3.64; words recognition: *t* = 11.90, *p* < 0.001, *d* = 2.59), young adults (pictures recognition: *t =* 15.45, *p* < 0.001, *d* = 2.98; words recognition: *t* = 12.92, *p* < 0.001, *d* = 2.39), and middle-aged adults (pictures recognition: *t* = 10.12, *p* < 0.001, *d* = 2.42; words recognition: *t* = 9.35, *p* < 0.001, *d* = 2.30).

## Discussion

We found interesting results in the current study. For all age groups, recognition scores for both pictures and words in the baseline test were significantly higher than IB experiment. In both the baseline recognition experiment and the IB experiment, adolescents performed better than young adults, who performed better than middle-aged adults. Participants across all age groups achieved higher scores under the congruency condition in comparison with incongruency condition between unattended stimuli and attended stimuli. However, although participants performed a little higher score under picture recognition than words recognition, the differences didn’t reach to statistical significant.

In baseline test, all the words and pictures were attended stimuli, while in the IB experiment, all the recognition stimuli were unattended stimuli. According to hybrid perceptual load model (Cartwright-Finch and Lavie, 2007), when the main task involved high level of perceptual load, focusing attention on the primary tasks prevented the perception of task-irrelevant stimuli. In the current IB experiments, on account of counting repetitive words or pictures occupied more of the attention capacity, little attention capacity spilled over automatically to process unattended stimuli, therefore, the recognition scores for unattended stimuli were lower than baseline recognition.

In the current study, we adopted the IB paradigms that differ from previous studies and found age-related decreased detection for the unattended stimuli from adolescents to young adults and middle-aged adults. As introduced earlier, a recent study found decreased detection accuracy for unattended objects with increasing age (Stothart et al., 2015). Two recent studies consistently showed that older adults presented decreased detection of unattended stimuli than young adults (Graham and Burke, 2011; Horwood and Beanland, 2016). The current study extended previous findings and found that participants presented IB rate increases from the adolescents.

As mentioned in the introduction, although there were two popular theoretical models were proposed to explain the IB results: the attention capacity models and the inhibition deficit model. We had reasons to believe that the attention capacity models were more appropriate for explaining our age-related results and predicted greater IB in middle-aged adults than young adults.

The results verified this hypothesis. The adolescents and young adults presented better explicit capture processing, which could in part be supported by their higher baseline recognition scores, and then, more of the unattended information could be processed by them than the middle-aged adults. Previous studies also found cognitive abilities, especially the fluid intelligence decreased from the young adults or even the later adolescents, could be a factor. Researchers found age-related decreased in visuo-spatial processing, perceptual speed, reasoning, and memory but an inverted-U shaped lifespan trajectory of the vocabulary processing with a peak age of 70 years (Salthouse, 2010). Craik and Bialystok (2006) systematically reviewed the cognition across the lifespan, and they proposed that cognitive control peaked in the late adolescence and early 20s while it declined with aging. Young adults showed significantly lower recognition performance for the unattended pictures and words than adolescents. The reasons were not clear, however, and a recent task switch study might lent some help for understanding the findings. Task switch was an important component of cognitive control. In a gender and emotion switch task, researchers found adolescents (approximately 16.5 years) showed the fastest response times for emotion, gender, and switching blocks than young and middle-aged adults; meanwhile, they also showed less switch costs than young and middle-aged adults (Reimers and Maylor, 2005). According to these findings, we speculated that adolescents may had more resources to process the unattended stimuli.

Although interaction between age and congruency was significant, which suggested the effect of congruency on IB would be distinct in three age groups, we found that participants across all age groups presented higher recognition scores for the unattended pictures and words under congruency condition in comparison with incongruency condition. The contingent attention capture theory considered that a stimulus could capture attention only with the stimulus itself or its characteristics would be included in the participants’ attentional set, while the participants’ attentional set was decided largely by the current primary task (Folk et al., 1992). According to the sustained IB experimental results, Most et al. (2005) extended this theory and considered that unattended stimuli could automatically attract transient, implicit attention, while participants’ own attentional set determined the additional attention directed toward the stimuli. Therefore, when the unattended stimuli had similar characteristics to the attended objects, the detection rate would increase. The current results supported these findings. In the congruency condition, the superimposed pictures and the words shared the same semantic information, while in the incongruency condition, they represented different semantic information. Many studies also provided consistent findings. Mack and Rock (1998) also suggested that the participants could detect the unattended stimuli if the stimulus was the observers’ name or happy faces. However, the unattended familiar words, another person’s name, a neutral face or colorful dots could not capture observers’ attention. Researchers concluded that the meanings of the unattended stimuli were the key factor in capturing attention (Mack and Rock, 1998). With the dynamic IB task, researchers found that both young and older adults presented higher IB rates for white unattended stimuli when they attended the black target while ignoring white distractors (Horwood and Beanland, 2016). In an IB study combined with a flanker task, participants were asked to judge the central letter while ignoring the lateral letter case (Buetti et al., 2014). In the fourth trial, an unattended square or letter appeared on the screen. Participants reported seeing 43% (18 out of 42 participants) and 81% (34 out of 42 participants) of the unattended square or letter. They further explored the flanker-like congruency effects in the letter group (unattended stimuli is letter) because 81% of them observed the unattended stimuli. They found that participants performed correctly and had a mean response time of 1743 ms (*SD* = 839 ms) when unattended stimuli were congruent with the central letter, while participants only made 31% correct responses and had a mean response time of 2359 ms (*SD* = 1854 ms) when unattended stimuli were incongruent with the central letter. In a driving-related IB task, researchers considered whether participants could observe the expected stimuli such as a pedestrian or an animal in the country or city-related driving scenarios. The results showed that participants presented higher detection percentages in the city scenarios, and congruent stimuli were seen more than incongruent stimuli (Pammer and Blink, 2013).

In the current study, there were no significant recognition differences for unattended pictures or words in adolescents and young adults. We only found that middle-aged adults achieved higher recognition scores for unattended pictures in comparison with unattended words. Devue et al. (2009) adopted the photographic stimuli to study the IB effects. They found that participants would detect more of the unattended pictures of faces in comparison with pictures of common objects (lemon, strawberry, potato, or pear) and pictures of inverted faces. Mack and Rock (1998) found that participants could capture unattended natural scenes. One recent study suggested that pictures were processed more readily than words when attended; the reason might be because pictures could maintain more direct access to semantic representations compared to words, even under conditions in which pictures were actively ignored (Walker et al., 2017). To our knowledge, there was no study that directly compared the IB rate for the unattended pictures and words; therefore, the present results need to be further investigated.

There were some limitations in the current study. First, because the preliminary results showed that older adults presented very low recognition performance under IB condition, we did not include older adults. Second, Hannon and Richards (2010) found that the attention span while not visually working memory capacity contributed to the experiences of IB. In the current study, we did not include other cognitive tasks such as working memory, inhibition, or attention span tasks, while these tasks were helpful in explaining the IB results. Third, theoretically, it would be great if the same participants performed the baseline recognition test and the IB experiments, and then the baseline performance could be taken as a covariate in the IB data analyses, however, on account of participants might guess or realize the unattended recognition task, the main task could be affected. Therefore we recruited different participants to participate in the baseline recognition test and IB experiments. Despite these limitations, the current results proved an age-related decrease in unattended stimuli recognition with relatively large samples.

## Conclusion

Participants across groups of adolescents, young adults and older adults presented significantly lower recognition scores for unattended pictures and words in comparison with the baseline recognition. For unattended pictures and words recognition, the current results showed decreased recognition scores from the adolescents to young and middle-aged adults, and all participants presented better recognition scores when attended stimuli and unattended stimuli were consistent. The current findings partly supported the attentional capacity models of IB.

## Author Contributions

H-HL conceived the idea, collected the data, and wrote the manuscript.

## Conflict of Interest Statement

The author declares that the research was conducted in the absence of any commercial or financial relationships that could be construed as a potential conflict of interest.
